# Comprehensive Analysis of Differential Immunocyte Infiltration and Potential ceRNA Networks Involved in the Development of Atrial Fibrillation

**DOI:** 10.1155/2020/8021208

**Published:** 2020-09-19

**Authors:** Jiafeng Wu, Huiming Deng, Qianghua Chen, Qiang Wu, Xiaolong Li, Siwei Jiang, Fengxin Wang, Fuyin Ye, Langhui Ou, Hong Gao

**Affiliations:** ^1^Department of Cardiology, The Third People's Hospital of Shenzhen, Shenzhen 518114, China; ^2^Department of General Surgery, Zengcheng District People's Hospital of Guangzhou, Guangzhou 511300, China

## Abstract

This study is aimed at identifying potential molecular mechanisms and candidate biomarkers in the left atrial regions for the diagnosis and treatment of valvular atrial fibrillation (VAF). Multibioinformatics methods, including linear models for microarray analysis (LIMMA), an SVA algorithm, CIBERSORT immune infiltration, and DNA methylation analysis, were employed. In addition, the protein-protein interaction (PPI) network, Gene Ontology (GO), and molecular pathways of differentially expressed genes (DEGs) or differential methylation regions were constructed. In all, compared with the normal rhythm group, 243 different mRNAs (29 downregulated and 214 upregulated) and 26 different lncRNAs (3 downregulated and 23 upregulated) were detected in the left atrium (LA) of atrial fibrillation (AF) patients, and the neutrophil and CD8^+^ T cell were infiltrated. Additionally, 199 different methylation sites (107 downregulated and 92 upregulated) were also identified based on DNA methylation analysis. After integration, ELOVL2, CCR2, and WEE1 were detected for differentially methylated and differentially transcribed genes. Among them, WEE1 was also a core gene identified by the competing endogenous RNA (ceRNA) network that included WEE1-KRBOX1-AS1-hsa-miR-17-5p, in VAF left atrial tissue. We combined the DNA methylation and transcriptional expression differential analysis and found that WEE1 (cg13365543) may well be a candidate gene regulated by DNA methylation modification. Moreover, KRBOX1-AS1 and WEE1 can compete endogenously and may mediate myocardial tissue infiltration into CD8^+^ T cells and participate in the AF process.

## 1. Introduction

Atrial fibrillation (AF) is the most common arrhythmia that presents in clinical practice. With the development of an aging population, its incidence has been increasing year by year. Studies suggest that the progression of AF is closely related to hypertension, heart failure, and myocardial infarction, which can significantly increase the risk of stroke and sudden cardiac death. The mechanism of development and progression of AF is closely related to myocardial electrophysiology and structural disorders [1]. In terms of electromechanical disorders, an irregular RR interval and P wave and disordered electrical activity can lead to ineffective atrial contraction and further structural (and electrical) changes in the atrium and promote the malignant progression of AF [2]. However, no consensus has been reached on the exact mechanisms and pathophysiological changes involved in AF, but it is certain that AF is one of the final manifestations of the pathological process of multiple diseases, and its occurrence and maintenance cannot be explained by a single mechanism [3, 4]. Due to long-term hemodynamic changes and long-term anticoagulant or irregular medication, the risk of cardiovascular and cerebrovascular complications and malignant events in patients significantly increased [3]. Therefore, clarifying the occurrence and development mechanisms underlying AF is of great significance for predicting the early occurrence of AF and identifying the early therapeutic targets to limit or prevent its progression. As a newly developed technology, genomics and DNA sequencing have greatly expanded the research scope of medical molecular biology. At present, the genetic studies based on the integrated analysis of the transcriptome and genome-wide methylation have opened up new horizons in our understanding of disease progression, phylogeny, inflammation, and immune responses. The techniques further allow the precision and individualized diagnosis and treatment of diseases diagnosed by modern medicine [2, 4]. The present study is aimed at screening differential genes and methylated regions in the atrial tissue of AF patients, building a network of AF-related molecular biological mechanisms, and exploring key regulatory factors in its progression to facilitate the discovery of diagnostic and therapeutic targets related to the development of valvular atrial fibrillation (VAF), which are of great research significance [3].

## 2. Methods

### 2.1. Data Screening and Acquisition

After retrieval, we downloaded the original data of the 3 groups of microarray chips, namely, GSE79768, GSE41177, and GSE14975 from the GEO database, and the original data of the methylated chip GSE62727. Among them, the chip platforms of GSE79768 [5], GSE41177 [6], and GSE14975 [7] are GPL570 [HG U133_Plus_2] Affymetrix Human Genome U133 Plus 2.0 Array platform (Affymetrix, Santa Clara, CA, US). GSE79768 contains 26 left and right atrial samples (7 patients with VAF and 6 patients with sinus rhythm (SR)), GSE14975 contains 10 left atrial tissue samples (5 patients with VAF and 5 patients with SR), and GSE41177 contains 19 left atrial samples (16 patients with VAF and 3 patients with SR). The GSE62727 methylation chip matches the GPL13534 Illumina Human Methylation 450 BeadChip (Human Methylation 450_15017482) platform and contains 11 left atrial samples (7 patients with VAF and 4 patients with SR) [8].

### 2.2. Data Analysis of Microarray Chips

The data processing flows of GSE79768, GSE41177, and GSE14975 chips were as follows: (1) the CEL fluorescence intensity values were read; (2) quality control; (3) strength value background processing and probe annotation; (4) log2 conversion, processing missing values, and linear models for microarray data (LIMMA) for difference statistical analyses [9]; (5) elimination of interchip batch effects followed by data merging; and (6) principal component analysis (PCA) was then conducted to view data structures.

In addition, the data processing strategies included the following: (1) referring to the study of Troyanskaya et al., the k-nearest neighbor (KNN) algorithm was used to process probe missing values [10] to ensure the robustness of the data; (2) three different studies of GSE79768, GSE41177, and GSE14975 have potential batch effects and data heterogeneity due to experimental sites and technical operations. Referring to the research of Leek et al., they used the algorithm of variable substitution and estimation of the SVA function to adjust the batch effect between high-throughput data [11]; (3) robust multiarray average (RMA) was used for background adjustment, normalization, and logarithmic transformation of probe expression values. If multiple probes matched with the same gene, the average value was considered as the gene expression value [10]; (4) further identification of differentially expressed lncRNAs with reference to the HGNC database (HUGO Gene Nomenclature Committee) and the NetAffx annotation file [12]; and (5) LIMMA was performed between the intergroup sequencing data, and the differentially expressed genes were identified by cross-checking, and then the statistical *P* value of the false discovery rate (FDR) was adjusted by the Benjamini-Hochberg method to calculate the expression fold change (FC) (log2FC > 1 and corrected *P* < 0.05 for differential genes) [13].

### 2.3. Gene Set GO and Pathway Enrichment Analysis

Gene Ontology (GO) and Kyoto Encyclopedia of Genes and Genomes (KEGG) enrichment pathway analysis results were obtained based on the MetaScape gene annotation and retrieval platform (http://metascape.org/gp/index.html) [13].

### 2.4. DNA Methylation Data Analysis

The GSE62727 methylation original IDAT file processing flow was as follows: (1) the raw data was read, (2) data preprocessing and data quality control, (3) matching with the reference genome, (4) methylation level calculation, (5) differential methylation site and segment analysis, and (6) differential gene and CpG island notes. Among them, the proportion of oligonucleotides whose beta value reaction could match the methylated sequence in the methylation analysis was the methylation rate of the sequence; the *M* value was the log conversion value based on the beta value, which eliminated interference by the chip probe [14].

### 2.5. Immunoinfiltration Analysis

CIBERSORT (https://cibersort.stanford.edu/) is an immune subtype infiltration calculation algorithm based on linear support vector regression [15]. Users can estimate the infiltration level of each subtype by chip expression profiling and RNA-seq expression data. The parameters involved in the present study included the following: (1) an RMA algorithm corrected the gene expression value; (2) the deconvolution (Perm) parameter was 1,000; (3) the differential subtype was identified as *P* < 0.05.

### 2.6. Network Construction of Competing Endogenous RNAs

The network construction of competing endogenous RNAs (Cennis) was based on miRcode (http://www.mircode.org/) [16], miRDB (http://mirdb.org/) [17], miRTarBase (http://mirtarbase.mbc.nctu.edu.tw/php/index.php) [18], and TargetScan (http://www.targetscan.org/mamm_31/) [19] databases which were used to build a further competing endogenous RNA (ceRNA) network.

Among them are as follows: (1) predicting differential miRNAs targeted by lncRNAs through a highly conserved microRNA family of miRcode databases; (2) predicting mRNAs targeted by candidate miRNAs through miRDB, miRTarBase, and TargetScan databases, wherein each predicted value needed to satisfy a matching number ≥ 2; (3) the final ceRNA network was constructed by taking the differential mRNA and miRNA-targeted mRNAs obtained in the present study.

## 3. Results

### 3.1. Data Acquisition and Preprocessing

**Figure 1:**
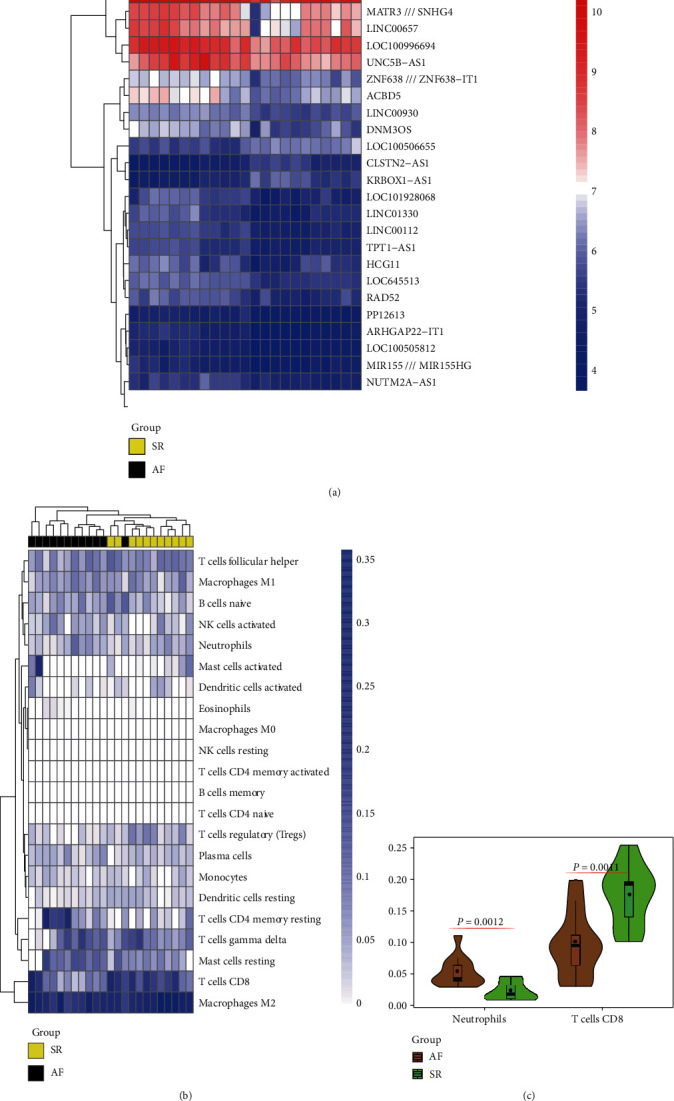
Heat map of the difference in lncRNA expression and immunocyte infiltration in atrium specimen cross-comparison of patients with AF and SR. (a) The differentially expressed lncRNA among the SR and AF LA tissues. (b) The immunocyte infiltration level of in SR and AF LA tissues. (c) The hub immunocytes were detected.

After integration, 3 sets of chips (GSE79768, GSE41177, and GSE14975), including 14 left atrial samples of normal heart rhythm patients and 28 left atrial samples of VAF, were analyzed. The difference analysis results suggested that compared with the normal group, there were 243 different mRNAs (29 downregulated and 214 upregulated) and 26 different lncRNAs (3 downregulated and 23 upregulated) in the left atrial myocardium in the VAF group. Among them, differential lncRNA expression is shown in Figure 1(a).

### 3.2. Immunoinfiltration Analysis

The overall immune infiltration map of left atrial tissue in patients with AF is shown in Figure 1(b), in which subtypes, such as T cells, macrophages, and B cells, have significantly infiltrated the cardiac tissue. Compared with the normal rhythm group, the degree of infiltration of left atrial neutrophils and CD8^+^ T cells into the atrial fibrillation group was significantly increased (neutrophil, *P* = 0.0012; CD8^+^ T cells, *P* = 0.001) (Figure 1(c)). Therefore, neutrophils and CD8^+^ T cells may be potential core cell subtypes involved in driving the progression of AF disease.

### 3.3. DNA Methylation Data Analysis

**Figure 2:**
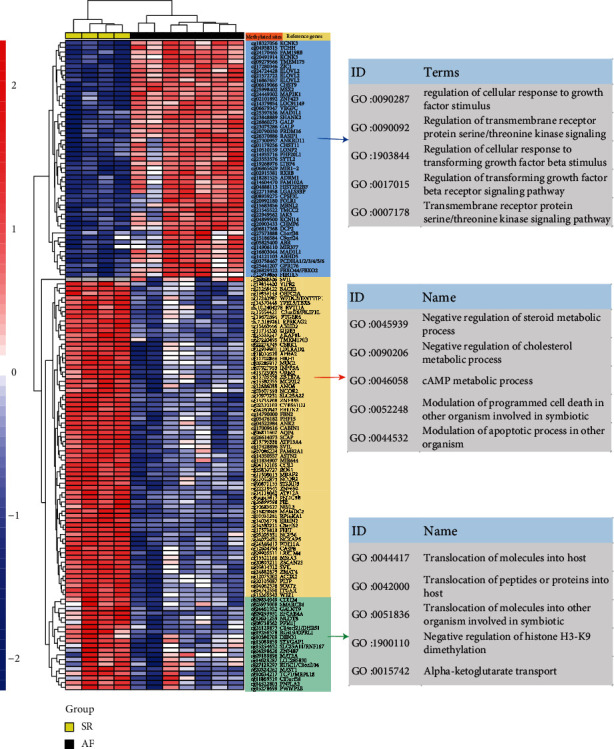
The detection of differential methylation regions and identification of Gene Ontology (GO) enrichment for the comparisons between AF and SR. This figure presents the results of differential methylation sites and GO function enrichment for referenced genes. Red represents higher expression; blue represents lower expression.

The 450 BeadChip methylation chip contains 96% of the methylation sites of the genome and is the current mainstream chip for methylation analysis. Compared with the SR group, there were 199 different methylation sites in VAF left atrial tissue, 107 of which were downregulated and 92 upregulated. Among them, differential methylation sites were closely related to pathway enrichment such as GO:0090287—regulation of the cellular response to growth factor stimuli (*P* = 1.53^−06^, *n* = 7), GO:0010894—negative regulation by steroid biosynthetic process (*P* = 6.13^−05^, *n* = 3), and GO:0044417—translocation of molecules into the host (*P* = 1.13^−03^, *n* = 1) (Figure 2).

**Figure 3:**
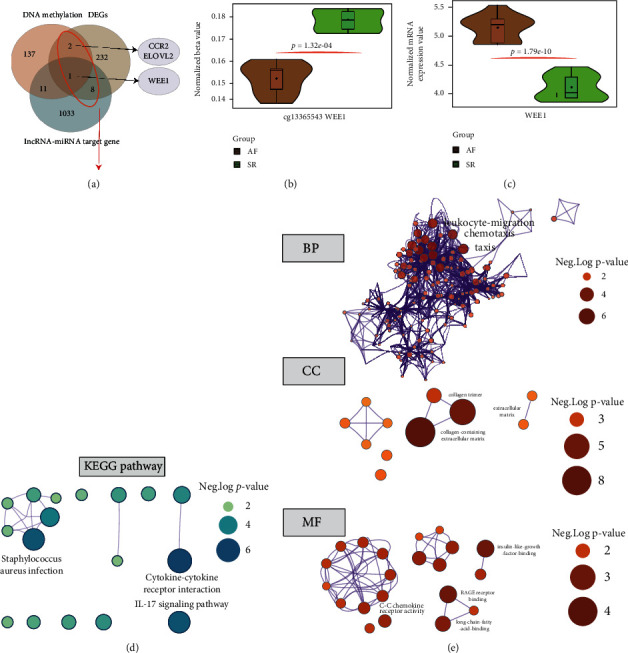
The identification of hub genes and gene function enrichment analysis for the integrative analysis between transcriptome and DNA methylation analysis. (a) The overlap detection among the differentially expressed lncRNA-miRNA target gene, DNA methylation gene, and differential expressed mRNA. (b, c) The expression analysis of hub methylation site and mRNA. (d) The KEGG pathway enrichment based on GSEA analysis. The size of the dots represents the neg. (e) The results of GO function enrichment for differentially expressed genes. The size of the nodes represents the value of neg.

After annotation according to methylation sites, the three genes in the present study were differentially methylated and differentially transcribed (ELOVL2, CCR2, and WEE1) (Figure 3(a)). Among them, WEE1 was also a core gene identified by the ceRNA network of differential lncRNA-miRNA-mRNA and could be used as a candidate regulator in the present study.

We further analyzed and visualized the methylation site and gene expression values corresponding to the WEE1 gene and found that the methylation site cg13365543 was decreased by the AF group and at the WEE1 transcriptional expression level was upregulated, suggesting the transcription of the gene regulated by DNA methylation modification (Figures 3(b) and 3(c)).

### 3.4. Gene Ontology Functions and Pathway Enrichment Analysis

According to MetaScape database analysis, AF-related differential genes were mainly related to hsa04060: cytokine-cytokine receptor interaction (*P* = 0.001, *n* = 9), hsa04657: IL-17 signaling pathway (*P* = 0.0016, *n* = 5), and hsa05150: *Staphylococcus aureus* infection (*P* = 0.002, *n* = 4) (Figure 3(d)). Regarding GO enrichment, differential gene BP enrichment mainly included GO:0006935—chemotaxis (*P* = 2.51^−06^, *n* = 20), GO:0042330—taxis (*P* = 2.51^−06^, *n* = 20), and GO:0050900—leukocyte migration (*P* = 5.01^−06^, *n* = 17); CC is mainly enriched in GO:0062023—collagen-containing extracellular matrix(*P* = 1.0^−08^, *n* = 19), GO:0031012—extracellular matrix (*P* = 1.0^−08^, *n* = 20), and GO:0005581—collagen trimer (*P* = 1.58^−04^, *n* = 6); MF is closely related to GO:0005520—insulin-like growth factor binding (*P* = 1.26^−04^, *n* = 4) and GO:0036041—long-chain fatty acid binding (*P* = 2.51^−04^, *n* = 3) (Figure 3(e)).

### 3.5. ceRNA Network Construction

**Figure 4:**
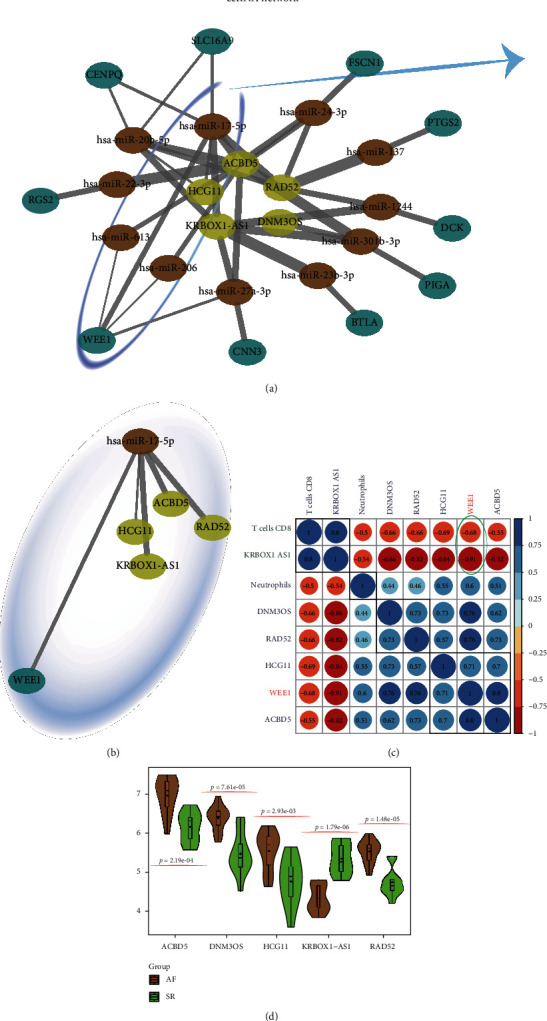
The competing endogenous RNA (ceRNA) network construction and relationship detection between them and immune cells. (a, b) Based on miRcode, miRDB, miRTarBase, and TargetScan databases, a complex ceRNA network was constructed and the core regulator, including WEE1-KRBOX1-AS1-hsa-miR-17-5p, in valvular AF left atrial tissues also detected. (c) The collinearity of the candidate mRNA, lncRNA, and immunocyte infiltrations. Each cell represents the Pearson correlation level between the row and column corresponding genes; the expression levels were presented in (d).

By integrating the miRcode, miRDB, miRTarBase, and TargetScan databases, an AF-related ceRNA was a network constructed which is shown in Figure 4(a). Among them, lncRNAs such as HCG11, KRBOX1-AS1, ACBD5, and RAD52 may compete with WEE1 for hsa-miR-17-5p (Figure 4(b)). Compared with the SR group, ACBD5, DNM3OS, HCG11, and RAD52 in these lncRNAs were significantly elevated in the AF group (all *P* < 0.05), while KRBOX1-AS1 was decreased in the AF group (*P* < 0.05) (Figure 4(d)). Through the Pearson correlation statistics and cluster analysis, we found that the KRBOX1-AS1 expression level was highly correlated with CD8+ T cell infiltration (Pearson coefficient 0.80; *P* = 5.32^−06^) but negatively correlated with WEE1 (KRBOX1-AS1 vs. WEE1: Pearson coefficient -0.91, *P* = 0.035; T cells CD8^+^ vs. WEE1: Pearson coefficient -0.68; *P* = 6.83^−04^) (Figure 4(c)).

## 4. Discussion

The occurrence and maintenance of AF are complex biological processes and are two of the ultimate manifestations of many cardiovascular diseases. From the perspective of pathophysiology, it can be considered that AF is the accumulation of pathogenic factors to a certain level over time, causing damage to atrial myocytes and leading to changes in genetic material, morphological structure, and dysfunction of atrial myocytes. In recent years, with the rapid development of various high-throughput technologies, researchers have been able to observe the modification and transcriptional changes in genetic material at the whole genome level. The study of genomic mapping and epigenomics can help researchers identify and annotate important functional regulatory elements in disease or morphological development and outline important gene regulatory regions in the complex process of human disease and organ development. Notably, a large-scale LA transcriptome analysis was performed by Tan's group that assessed transcriptome data from 156 subjects, and these results focused on cardiovascular function and development and organ morphology, and the interesting module was strongly associated with calcium signaling [20]. Similarly, based on a patient's history of AF, Deshmukh et al. accessed the left atrial appendage (LAA) differential expression of genes in 239 subjects that revealed a key role in the pathways of inflammation and oxidation and the cellular stress response associated with both AF susceptibility and persistence [21].

Recently, the integrated analysis and exploration of DNA methylation and RNA transcriptional expression have greatly improved the selection reliability and research efficiency of epigenetic regulation candidate genes, which is the mainstream strategy of disease research at the present time. Modification of DNA methylation is a special epigenetic covalent modification that mainly occurs at the cytosine-phosphate-guanine (CpG) dinucleotide. This covalent modification does not involve any direct alteration of the DNA sequence but plays an important role in regulating the transcriptional expression of the gene and stabilizing the chromatin conformation. It is generally believed that DNA methylation in the promoter region of a gene can cause a conformational change in DNA, thereby interfering with the recognition of a particular base sequence by the transcription factor, thus inhibiting the transcriptional expression level of the candidate gene.

In the present study, the maps of cytokine-cytokine receptor interaction have been identified in AF development and interpreted to support their central role in the regulation of the immunity/inflammatory response in physiological and pathological progress of cardiovascular disease. Increasing evidence suggests that the pathways involved in of cytokine-cytokine receptor interactions can stimulate or trigger an immune/inflammatory response via damage-associated molecular patterns (DAMPs) in “stressed” or “damaged” cells. The gene functional enrichment results of dysregulated transcripts presented significant changes in cytokine-cytokine receptor interactions and the chemokine signaling pathway, which shown a close relationship with myocardial ischemia/reperfusion injury as reported in Liu et al.'s study [22]. Gao et al. found that the cytokine-cytokine receptor interactions, apoptosis, and calcium signaling pathways were significantly associated with ionophore antibiotic-related cardiotoxicity via multiple molecular mechanisms [23].

We combined the DNA methylation and transcriptional expression differential analysis results and found that WEE1 (cg13365543) is likely to be a candidate gene regulated by DNA methylation modification. Moreover, KRBOX1-AS1 and WEE1 can compete with hsa-miR-17-5p endogenously and may mediate myocardial tissue infiltration into CD8^+^ T cells and participate in the AF process. Borden et al. found that the expression of WEE1 inhibits the cyclin B1/CDK1 complex and controls cell cycle reentry, thereby regulating the recovery of cardiac function in mice after myocardial infarction [24]. Similarly, Mohamed et al. found that WEE1 mainly had an influence on cell cycle factors and can play a role in the rerepair and proliferation of adult myocardial injury by inhibiting CDK1 and cyclin B [24, 25]. Kim et al. found that with the growth of the adult myocardium, the expression of WEE1 did not change but that expression inhibition occurred as a result of myocardial ischemia and hypoxic damage and further activated the functional activity of cyclin [24, 26]. KRBOX1-AS1 is a recently discovered lncRNA, and its function has been mainly reported to be associated with to the early development of severe early-onset eclampsia and preoperative chemotherapy responses in locally advanced rectal cancer [27, 28]. Ha et al. found that the KRBOX1-AS1 has been reported to play a key role in the proliferation and apoptosis of rectal cancer cells and thus affects preoperative radiation therapy in rectal cancer patients [28]. Additionally, KRBOX1-AS1 was shown to be significantly associated with inflammatory and immune response pathways and platelet and vascular development in severe early- or late-onset preeclampsia with intrauterine growth [27].

Bansal et al. found that CD8^+^ and CD4^+^T cells were involved in regulating the pathological process of progressive cardiac expansion, hypertrophy, and heart failure after permanent coronary artery ligation in mice and were closely associated with heart injury and remodeling [29]. Du et al. found that the infiltration and functional activation of CD8^+^ T cells was intimately linked with immune rejection after heart transplantation and the survival rate of transplanted hearts [30]. Similarly, both Ismahil and Ma's teams reported that CD8^+^ T cell infiltration promoted local expression and secretion of proinflammatory cytokines and chemokines, which are involved in myocardial defect injury or postinflammatory remodeling [31, 32].

In conclusion, the maps of cytokine-cytokine receptor interactions have been identified in LA samples, and they were interpreted to support the important role of LA in the development of AF. Subsequently, WEE1 (cg13365543) was identified as a candidate gene likely regulated by DNA methylation modification. Moreover, the network including KRBOX1-AS1 and WEE1 can compete with endogenous factors and may mediate myocardial tissue infiltration by CD8+ T cells and thus participate in the mechanisms underlying AF. Our results strongly suggest that these mechanisms are involved in the pathogenesis of inflammatory/immune responses, cell apoptosis, and membrane trafficking/ion transport. Thus, our findings have provided new insights into the pathogenetic mechanisms underlying AF.

## 5. Limitations

There were several limitations in our study. First, although this was a multiomics integration analysis, those external clinical traits (especially in clinical characteristics and sequencing-related technical errors of LA samples) were not used in our study. Second, there was no attempt to build an AF animal model in our study.

## Data Availability

Expression profile of GSE79768, GSE41177, GSE14975, and GSE62727 in the manuscript was downloaded from NCBI GEO (https://www.ncbi.nlm.nih.gov/gds).

## References

[1] January C. T., Wann L. S., Calkins H. (2019). 2019 AHA/ACC/HRS focused update of the 2014 AHA/ACC/HRS guideline for the management of patients with atrial fibrillation: a report of the American College of Cardiology/American Heart Association Task Force on Clinical Practice Guidelines and the Heart Rhythm Society. *Journal of the American College of Cardiology*.

[2] Morin D. P., Bernard M. L., Madias C., Rogers P. A., Thihalolipavan S., Estes N. A. M. (2016). The state of the art: atrial fibrillation epidemiology, prevention, and treatment. *Mayo Clinic Proceedings*.

[3] Magnani J. W., Rienstra M., Lin H. (2011). Atrial fibrillation: current knowledge and future directions in epidemiology and genomics. *Circulation*.

[4] Bapat A., Anderson C. D., Ellinor P. T., Lubitz S. A. (2018). Genomic basis of atrial fibrillation. *Heart*.

[5] Tsai F. C., Lin Y. C., Chang S. H. (2016). Differential left-to-right atria gene expression ratio in human sinus rhythm and atrial fibrillation: implications for arrhythmogenesis and thrombogenesis. *International Journal of Cardiology*.

[6] Yeh Y. H., Kuo C. T., Lee Y. S. (2013). Region-specific gene expression profiles in the left atria of patients with valvular atrial fibrillation. *Heart Rhythm*.

[7] Adam O., Lavall D., Theobald K. (2010). Rac1-induced connective tissue growth factor regulates connexin 43 and N-cadherin expression in atrial fibrillation. *Journal of the American College of Cardiology*.

[8] Zhou J., Gao J., Liu Y. (2014). Human atrium transcript analysis of permanent atrial fibrillation. *International Heart Journal*.

[9] Ritchie M. E., Phipson B., Wu D. (2015). Limma powers differential expression analyses for RNA-sequencing and microarray studies. *Nucleic Acids Research*.

[10] Troyanskaya O., Cantor M., Sherlock G. (2001). Missing value estimation methods for DNA microarrays. *Bioinformatics*.

[11] Leek J. T., Johnson W. E., Parker H. S., Jaffe A. E., Storey J. D. (2012). The sva package for removing batch effects and other unwanted variation in high-throughput experiments. *Bioinformatics*.

[12] Braschi B., Denny P., Gray K. (2019). Genenames.org: the HGNC and VGNC resources in 2019. *Nucleic Acids Research*.

[13] Tripathi S., Pohl M. O., Zhou Y. (2015). Meta- and orthogonal integration of influenza “OMICs” data defines a role for UBR4 in virus budding. *Cell Host & Microbe*.

[14] Gevaert O. (2015). MethylMix: an R package for identifying DNA methylation-driven genes. *Bioinformatics*.

[15] Newman A. M., Liu C. L., Green M. R. (2015). Robust enumeration of cell subsets from tissue expression profiles. *Nature Methods*.

[16] Jeggari A., Marks D. S., Larsson E. (2012). MiRcode: a map of putative microRNA target sites in the long non-coding transcriptome. *Bioinformatics*.

[17] Chen Y., Wang X. (2020). MiRDB: an online database for prediction of functional microRNA targets. *Nucleic Acids Research*.

[18] Chou C. H., Shrestha S., Yang C. D. (2018). MiRTarBase update 2018: a resource for experimentally validated microRNA-target interactions. *Nucleic Acids Research*.

[19] Agarwal V., Bell G. W., Nam J. W., Bartel D. P. (2015). Predicting effective microRNA target sites in mammalian mRNAs. *Elife*.

[20] Tan N., Chung M. K., Smith J. D. (2013). Weighted gene coexpression network analysis of human left atrial tissue identifies gene modules associated with atrial fibrillation. *Circulation. Cardiovascular Genetics*.

[21] Deshmukh A., Barnard J., Sun H. (2015). Left atrial transcriptional changes associated with atrial fibrillation susceptibility and persistence. *Circulation. Arrhythmia and Electrophysiology*.

[22] Liu Y., Li G., Lu H. (2014). Expression profiling and ontology analysis of long noncoding RNAs in post-ischemic heart and their implied roles in ischemia/reperfusion injury. *Gene*.

[23] Gao X., Peng L., Ruan X. (2018). Transcriptome profile analysis reveals cardiotoxicity of maduramicin in primary chicken myocardial cells. *Archives of Toxicology*.

[24] Borden A., Kurian J., Nickoloff E. (2019). Transient introduction of miR-294 in the heart promotes cardiomyocyte cell cycle reentry after injury. *Circulation Research*.

[25] Mohamed T. M. A., Ang Y. S., Radzinsky E. (2018). Regulation of cell cycle to stimulate adult cardiomyocyte proliferation and cardiac regeneration. *Cell*.

[26] Kim S. O., Katz S., Pelech S. L. (1998). Expression of second messenger- and cyclin-dependent protein kinases during postnatal development of rat heart. *Journal of Cellular Biochemistry*.

[27] Nevalainen J., Skarp S., Savolainen E. R., Ryynanen M., Jarvenpaa J. (2017). Intrauterine growth restriction and placental gene expression in severe preeclampsia, comparing early-onset and late-onset forms. *Journal of Perinatal Medicine*.

[28] Ha Y. J., Tak K. H., Kim C. W. (2017). PSMB8 as a candidate marker of responsiveness to preoperative radiation therapy in rectal cancer patients. *International Journal of Radiation Oncology • Biology • Physics*.

[29] Bansal S. S., Ismahil M. A., Goel M. (2017). Activated T lymphocytes are essential drivers of pathological remodeling in ischemic heart failure. *Circulation Heart Failure*.

[30] Du G., Yang N., Gong W. (2017). CD8^+^ effector memory T cells induce acute rejection of allogeneic heart retransplants in mice possibly through activating expression of inflammatory cytokines. *Experimental Cell Research*.

[31] Ismahil M. A., Hamid T., Bansal S. S., Patel B., Kingery J. R., Prabhu S. D. (2014). Remodeling of the mononuclear phagocyte network underlies chronic inflammation and disease progression in heart Failure. *Circulation Research*.

[32] Ma F., Feng J., Zhang C. (2014). The requirement of CD8^+^ T cells to initiate and augment acute cardiac inflammatory response to high blood pressure. *Journal of Immunology*.

